# Photoactivation of luminescence in CdS nanocrystals

**DOI:** 10.3762/bjnano.5.40

**Published:** 2014-03-25

**Authors:** Valentyn Smyntyna, Bogdan Semenenko, Valentyna Skobeeva, Nikolay Malushin

**Affiliations:** 1 Experimental Physics Department Odessa; I.I. Mechnikov National University; 2, Dvoryanska St., Odessa, UA-65026, Ukraine

**Keywords:** cadmium sulfide (CdS), luminescence, nanoparticles, short-wavelength emission band, surface modification

## Abstract

This paper presents the results of the research on the luminescence of cadmium sulfide nanocrystals (NCs) synthesized by colloid chemistry in a gelatinous matrix. The photostimulation of the short-wavelength emission band with λ_max_ = 480 nm has been detected. It is shown that the determining factor of the photostimulation effect is the adsorption of the water molecules on the surface of NC. The observed effect is explained by the recombination mechanism that is responsible for the short-wavelength emission band.

## Introduction

Semiconductor nanocrystals are an important class of materials due to the direct connection of their optical and electronic properties with the size of the particles. In particular the photoluminescence spectrum of high-quality nanocrystals extends from the UV to the IR spectral region due to the size-dependence of the energy spectrum. For that reason these materials are commonly used as luminophores in light emitting devices. A variety of luminescence colors can be produced from the same material by controlling the size of the nanocrystals. An important property of crystals that have a size in the nanometer-range and are isolated in a polymer matrix is a significant chemical activity of the surface. It is important to experimentally investigate the nanosystems with interphase boundaries between the nanocrystal and matrix and study the processes that occur at this interface. There are particularly important questions about the possibility of modifying the surface of the nanocrystals in order to obtain the required properties of the nanostructures. Factors that affect the adsorption processes occurring at the interface include the chemical composition of the matrix material and external influences, such as radiation and a thermal processing of nanostructures.

The luminescence properties of II–VI quatum dots (QDs) depend mostly on interfacial processes, which occur at the boundary between the QDs and the surrounding medium. This results in particular properties of luminescence of QDs such as photoinduced fluorescence enhancement (PFE) [1–5], intermittency or blinking of photoluminescence [6–8], and a blue- or red-shift of the exciton PL of nanocrystals [5,9]. Despite the large number of works devoted to the surface interactions in II–IV QDs [2–5,10] the mechanisms responsible for the interfacial processes and the mechanism of the amplification of the quantum yield of quantum dots are still a subject of active discussion and require further clarification [5]. As noted in [2–5,10–14] the properties of luminescence are strongly dependent on the technology of the quantum dots synthesis, nature of the coating agent, surrounding atmosphere, density of the quantum dots, and the radiation intensity.

Note that so far the unusual behaviour of the irradiation-induced QD luminescence has been observed either in QDs of CdSe or in nanocrystals of CdSe/ZnS/TOPO and has been mainly related to a single exciton emission band. Therefore, it is interesting to study the luminescent properties of quantum dots of other materials and other matrices that contain the QDs. Unlike [1–2,4–5] this paper investigates the luminescence of cadmium sulfide nanocrystals dispersed in a gelatinous polymer matrix, and the processes that occur at the interface boundary during irradiation of the nanocrystals in air and in vacuum. We also study the behavior of the entire spectrum of luminescence nanocrystals, including the long-wavelength band associated with defects.

## Experimental

The CdS nanocrystals used in these experiments were prepared by colloid chemistry of solutions of cadmium- and sulfursalts in an aqueous gelatin solution. Details of the sample preparation are described in [15]. The average radius of the cadmium sulfide nanocrystals was calculated from the optical absorption spectra according to the theory of interband absorption of light in nanoscale objects [16–17].

The luminescence of the samples was induced by a pulsed solid-state laser with a wavelength of λ = 355 nm. The luminescence was registered with a photoreceiver PMT-106 with a maximum spectral sensitivity in the range between 450 and 550 nm. Stationary and kinetic characteristics of the luminescence were recorded in air and in vacuum. In the latter case the samples were placed into a vacuum chamber at a pressure of 10^−2^ Torr. Measurements were carried out in the temperature range of 77–300 K. The temperature was measured by a differential copper–constantan thermocouple.

## Results and Discussion

**Figure 1:**
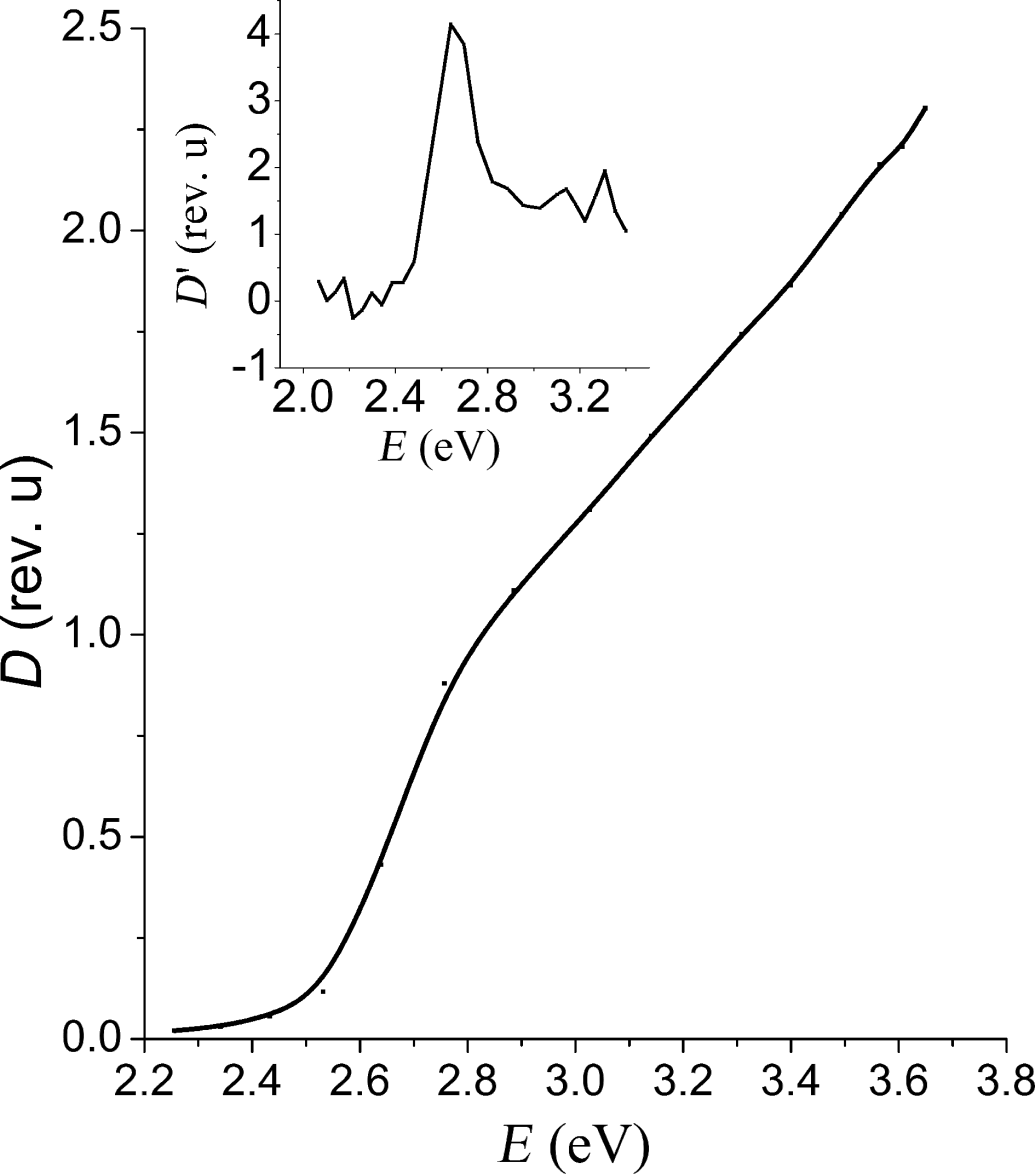
The absorption spectrum of the CdS nanocrystals. The inset shows the spectrum of the first derivative of the optical density of absorption.

The optical absorption spectrum of the investigated NC is shown in Figure 1. The long-wavelength absorption edge that corresponds to the first optical transition in CdS NC is shifted relative to the energy band gap of the bulk crystal CdS (2.5 eV) towards the short-wavelength region. The absence of the characteristic peaks that correspond to the individual transitions between discrete energy levels of the charge carriers indicates the dispersion of the size of the nanocrystals. The effective band gap of the nanocrystals was determined from the spectrum of the first derivative of the absorbance. The plot (inset in Figure 1) has a maximum corresponding to an energy of 2.64 eV. This value is adopted by us for the energy value of the first optical transition of the NC. The average radius of the CdS nanocrystals was estimated by using the expression for the size dependence of the energy of the optical transitions [16–17], and equals to 4.0 ± 0.2 nm.

**Figure 2:**
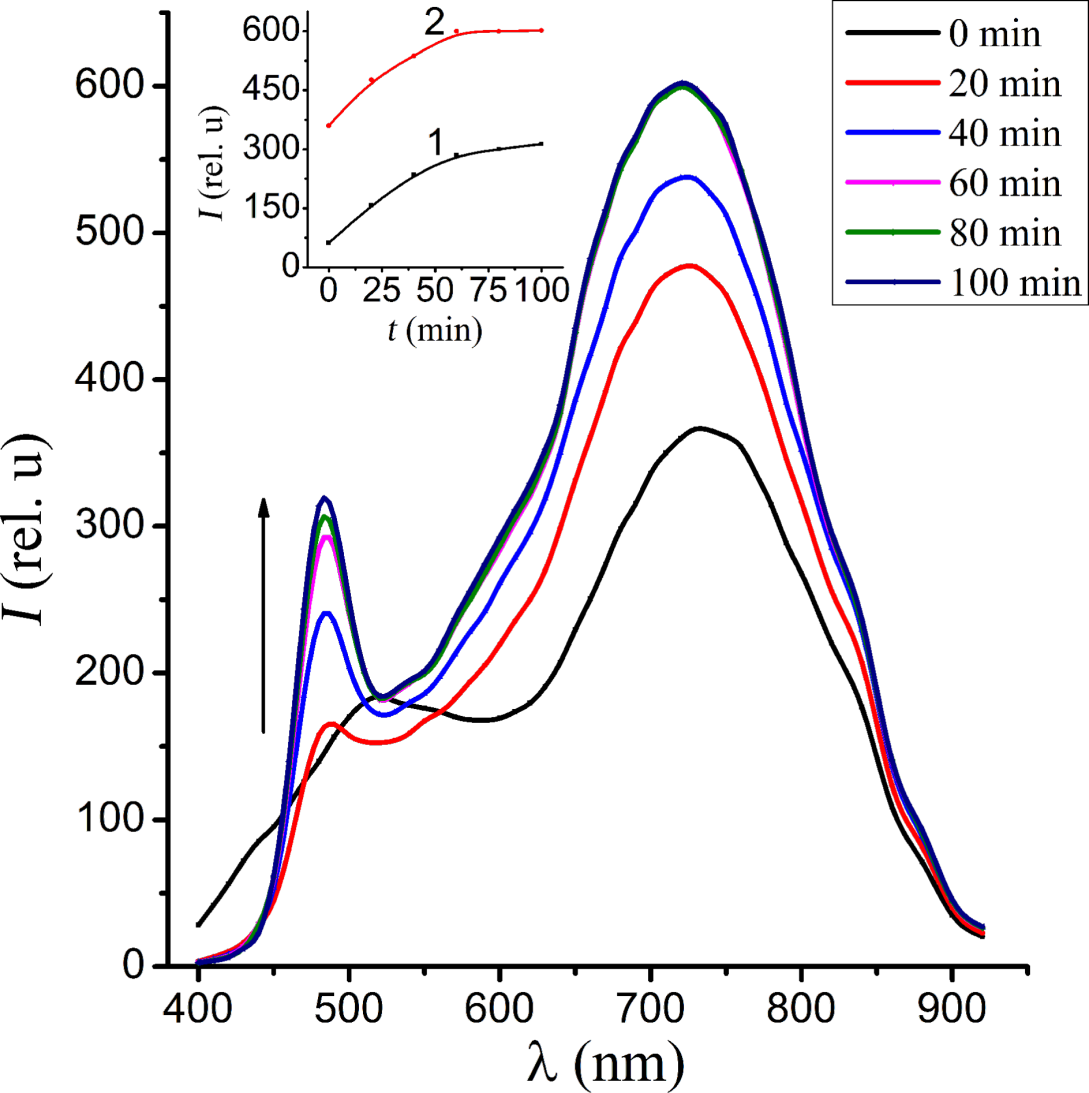
The luminescence spectra of CdS NC measured in air for a range of time intervals after the excitation irradiation. The inset shows the kinetics of the growth of the bands with intensity of λ_max_ = 480 nm (1) and λ_max_ = 720 nm (2).

The obtained CdS nanocrystals exhibit bright luminescence at room temperature. As can be seen from Figure 2, the luminescence spectrum measured in air at the initial moment of the excitation (curve denoted as "0 minutes") exhibits two wide bands of luminescence with λ_max_ = 520 nm (green) and λ_max_ = 720 nm (red). Further irradiation changes the contour of the luminescence band, namely the intensity of the band with λ_max_ = 720 nm increases, and the contribution of the green band reduces in the integrated spectrum. Furthermore a new band with λ_max_ = 480 nm appears. Note that here and in the subsequent figures the arrow indicates the direction of change of the luminescence intensity over time. The kinetics of the short-wavelength and long-wavelength luminescence bands are shown on the inset of Figure 2. Note that the first long-wavelength band is experiencing saturation. It is characteristic that such a state can be preserved while keeping the sample in darkness for the duration of several days. Overall this shows that the synthesized nanocrystals exhibit a photoinduced edge photoluminescence.

**Figure 3:**
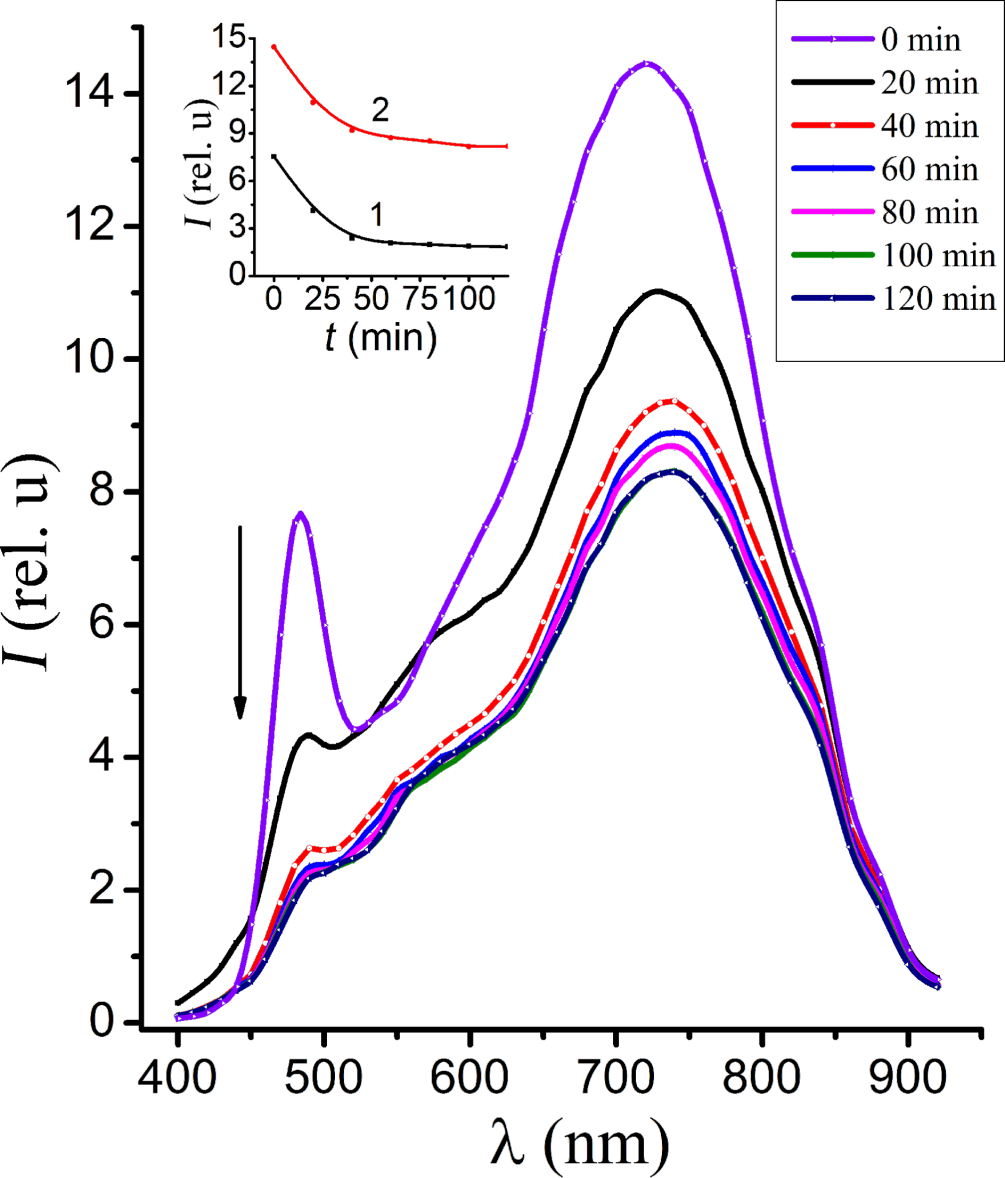
The luminescence spectra of CdS NC measured in vacuum for a range of time intervals after the excitation irradiation. The inset shows the kinetics of the decrease of the intensity of the bands λ_max_ = 480 nm (1) and λ_max_ = 720 nm (2).

**Figure 4:**
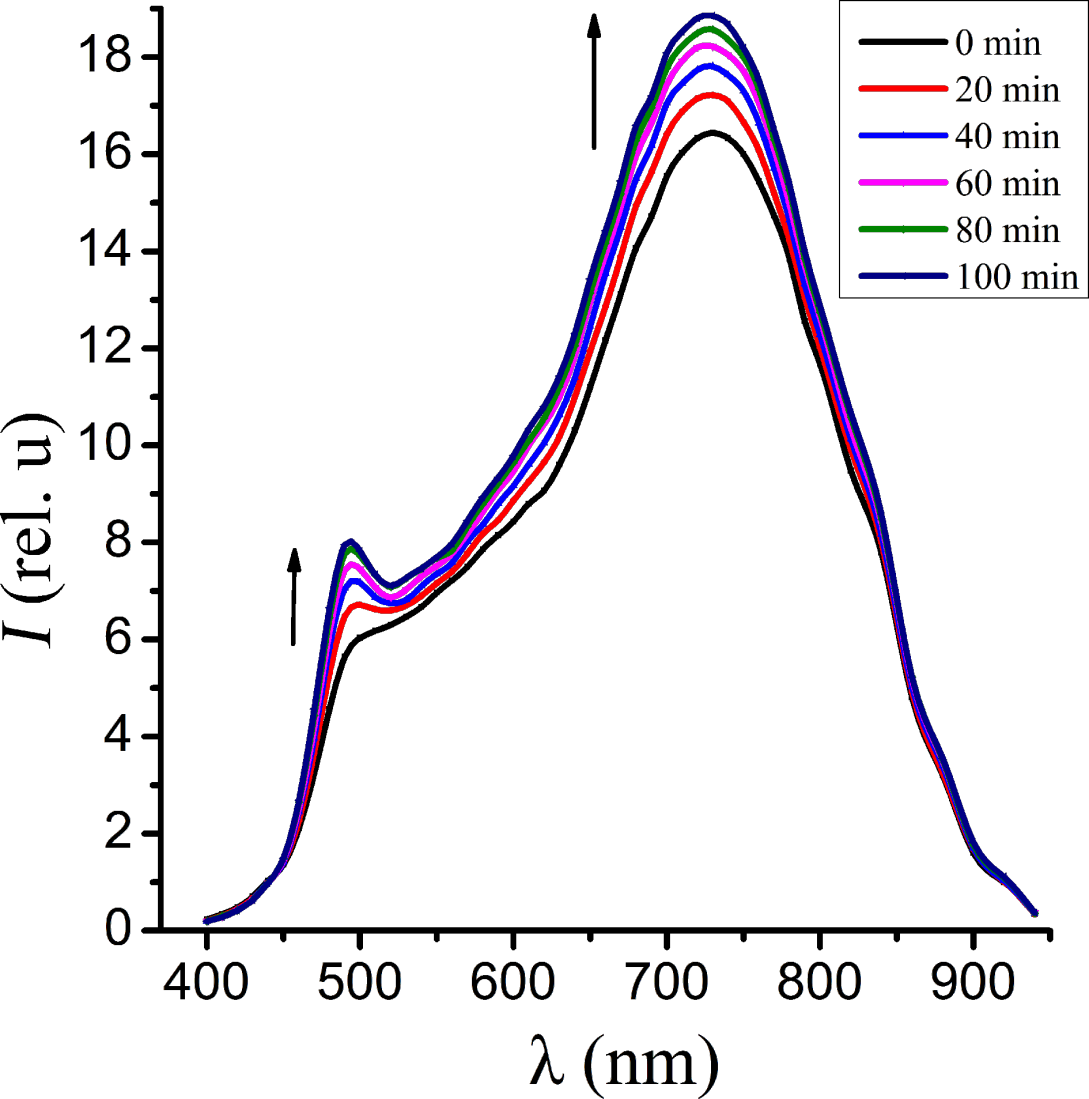
The luminescence spectra of CdS NC measured in air after being in vacuum, shown for a range of time intervals of the action of the exciting irradiation.

**Figure 5:**
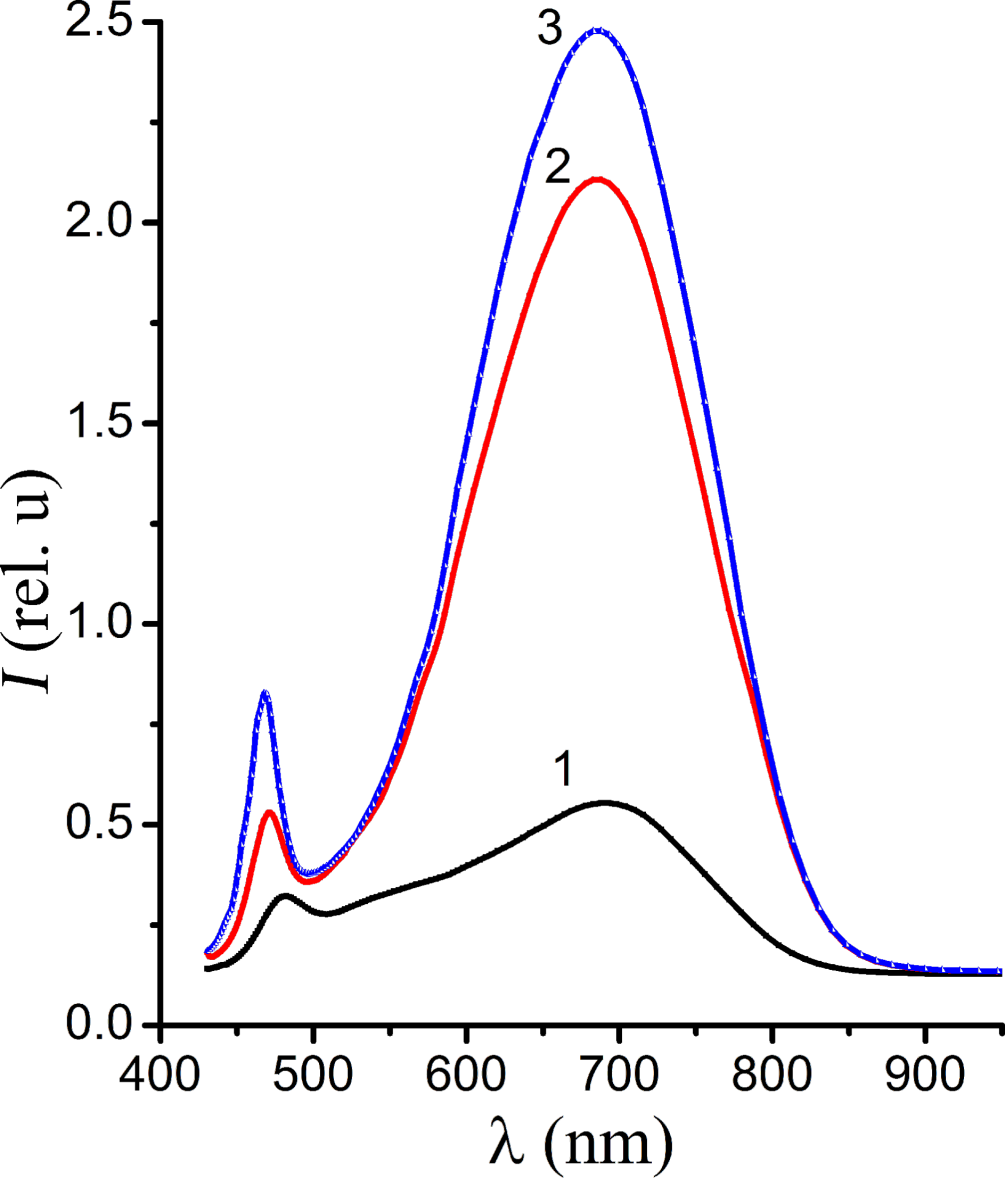
The luminescence spectra of CdS nanocrystals measured at temperatures of 300 K (1), 150 K (2) and 77 K (3).

In order to study the conditions that result in a photostimulation of the band radiation with λ_max_ = 480 nm we performed luminescence studies of the samples in vacuum. It has been found that the process observed in vacuum is reversed compared to the process observed in air. As is shown in Figure 3, at the initial moment two bands are registered at 480 nm and 720 nm. While the vacuum chamber is evacuated, the intensity of both bands reduces. After 100 min of irradiation essentially only one long-wavelength band is observed in the emission spectrum (Figure 4). When the chamber is vented again, the original luminescence spectra are recovered. In particular one can again observe the photostimulation of the short-wavelength band of the luminescence (see below in Figure 5).

The irradiation of cadmium sulfide nanocrystals in air or in humid atmosphere contributed to a high intensity of luminescence bands, which differs from the results described in [1]. In [1] an increase in the intensity of the exciton band is accompanied by a simultaneous decrease in the intensity of the "defective" luminescence band. In our experiments, we observed no shift of the maximum short-wavelength band luminescence and absorption edge to higher energies. This may indicate that the oxidative processes on the surface of the nanocrystals are not decisive in our samples. It is important to understand in which cases one can observe oxidative processes, which lead to a decrease in the size of the nanocrystals, a formation of the oxide shell on the surface of the nanocrystals and a quenching of the luminescence. In these cases one can observe processes of passivation of surface states, which contributes to an increase in the quantum yield of the luminescence.

In [5] a systematic investigation of the influence of humid argon and oxygen on the photoinduced amplification of the photoluminescence intensity of CdSe/ZnS quantum dots of is presented. It is suggested that this effect is the result of solvation of the charged states of the surface traps. Two competing processes may occur during the irradiation, when the samples are placed in a humid atmosphere. The dominance of either of them depends on the humidity level. It has been found experimentally that under low humidity, the main process is a reaction with oxygen, and at high humidity the photoinduced interaction of CdSe/ZnS QDs with water molecules prevails and an increase of the luminescence yield is observed.

Our samples differ from the CdSe/ZnS QDs, in which an amplification of photoindiced luminescence has been previously observed [1–5], because they are synthesized in an aqueous solution of gelatin. During the synthesis of cadmium sulfide nanocrystals gelatin plays the role of a growth stabilizer and a coating agent. Gelatin is a natural polymer that is capable of absorbing large amounts of moisture from the environment. It is evident that the cadmium sulfide nanocrystals are contained in the gelatin matrix, which contains a high concentration of water molecules. Thus, our results are consistent with the assumptions made in [5], in the sense that the effect of PFE is the result of photoactivation and stabilization of surface traps through adsorption of water molecules under conditions of high humidity.

According to [1,18–19], the nature of wavelength bands is associated with defects that are formed on the surface of nanocrystals. The short-wavelength band with λ_max_ = 480 nm has an exciton nature or it is related to the recombination of electrons and holes in the small centers in the valence band. That is, we observe luminescence that is caused by recombination processes in the bulk of the nanocrystals [20]. Note that for the distribution of nanocrystal sizes in our samples it is not always possible to distinguish between these two recombination mechanisms. In order to determine the luminescence mechanism of the short-wavelength band we have studied the temperature dependence of the luminescence. In order to prevent an exposure of the samples to ambient air at 300 K, the vacuum chamber was vented with vapour from liquid nitrogen. The CdS nanocrystals were photostimulated by laser irradiation at a wavelength of λ = 355 nm. The resulting luminescence spectra are shown in Figure 5. We observed an increase of the intensity of the long-wavelength and short-wavelength luminescence bands with the decrease in temperature.

**Figure 6:**
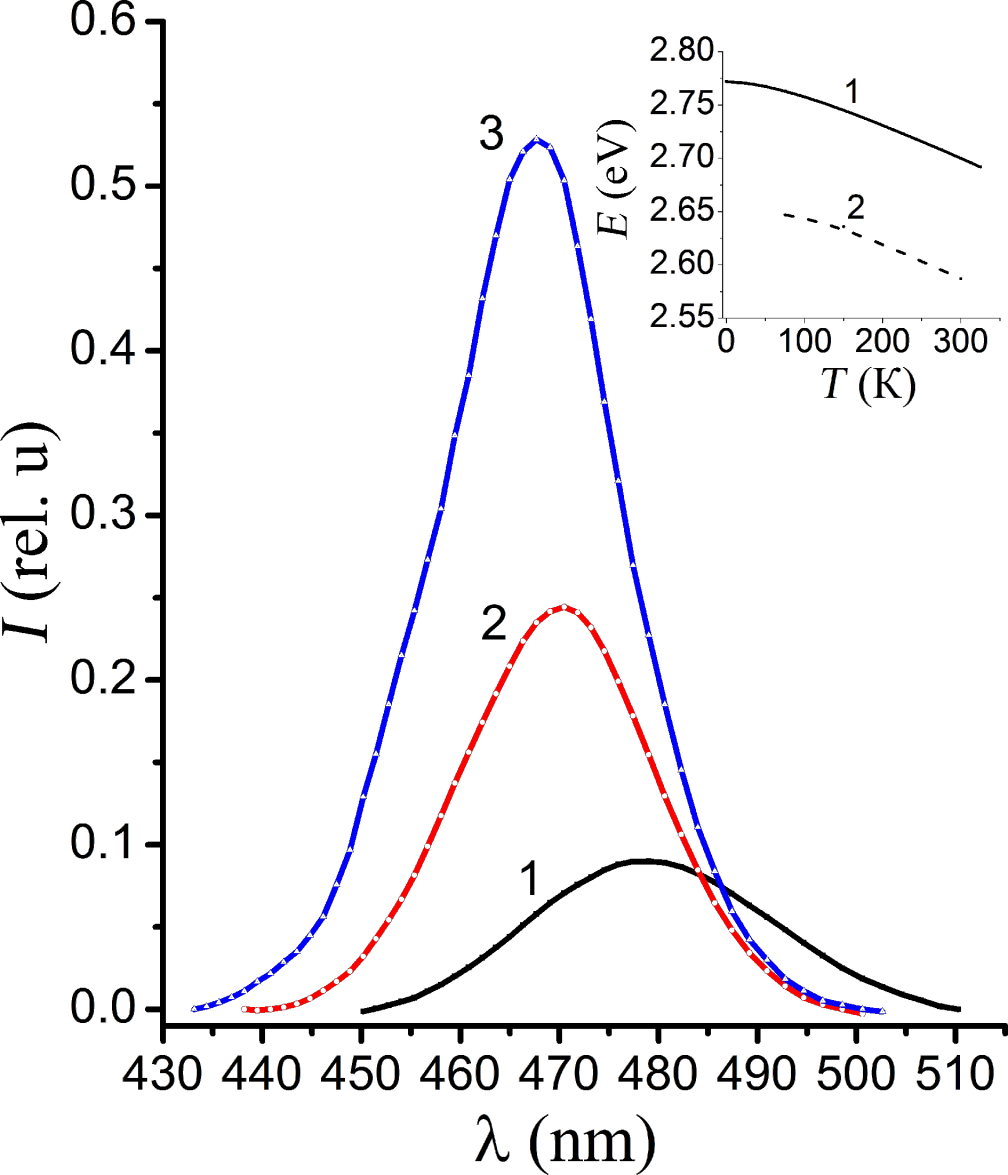
The edge luminescence spectra of CdS NCs at 300 K (1), 150 K (2) and 77 K (3). The inset shows the temperature dependence of the band gap of CdS NC (1) and the peak position of the edge luminescence of CdS NC (2).

The maximum of the long-wavelength band does not shift, but the maximum of short-wavelength band does shift towards lower wavelengths with decreasing temperature, as is illustrated in Figure 6. This indicates the edge nature of the short-wavelength luminescence band. In the inset of Figure 6 it is shown that with the changing the temperature the edge band of the photoluminescence shifts parallel to the band gap of NC. The energy separation between the band gap of the nanocrystal and the energy of the band maximum equals to 0.11 eV. It follows that the mechanism of recombination is the recombination of electrons that are captured by the small recombination centers with a depth of *E* = 0.11 eV and holes in the valence band.

## Conclusion

The experimental data presented in this paper shows a significant effect of interphase surface processes on the luminescence. Humidity of the air significantly influences the recombination processes in CdS nanocrystals. CdS nanocrystals considered in this study exhibited a photostimulation of the short-wavelength luminescence with λ_max_ = 480 nm. The determining factor of the photostimulation effect is the phenomenon of adsorption of water molecules on the surface of the nanocrystals. Hydroxy groups of the water molecules participate in the passivation of surface states, thereby reducing the contribution of non-radiative recombination. The mechanism of the recombination of the band with λ_max_ = 480 nm that is localized on the edge of the absorption spectrum is due to the recombination of the electrons that are captured in shallow donor centres with a depth of *E*_d_ = 0.11 eV with the holes in the valence band. This result is supported by the localization of the band at the edge of the absorption spectrum and the temperature dependence of the luminescence intensity in vacuum.
